# Examining the Associations of Risk Factors on Cognitive Performance in Latinos

**DOI:** 10.1002/alz70860_106900

**Published:** 2025-12-23

**Authors:** Diana Morales, Jacqueline Guzman, Yuliana Soto, Susan Aguiñaga

**Affiliations:** ^1^ University of Illinois at Urbana‐Champaign, Urbana, IL, USA; ^2^ Medical College of Wisconsin, Milwakee, WI, USA; ^3^ University of Illinois at Chicago, Chicago, IL, USA; ^4^ University of Illinois at Urbana Champaign, Urbana, IL, USA

## Abstract

**Background:**

Latinos in the U.S. are at high risk for Alzheimer's Disease and Related Dementias (ADRD) driven by modifiable factors like low education, poor sleep, inactivity, obesity, unhealthy diets, and diabetes. These factors often cluster, highlighting the need to study their combined effects on cognitive impairment. This study aims to explore the associations between clusters of ADRD risk factors and cognitive outcomes among Latinos.

**Method:**

This is a secondary analysis of a cross‐sectional study involving middle‐aged to older Latinos in Chicago and surrounding suburbs. Data were collected during two home visits in Spanish or English by bilingual staff, assessing demographics, chronic conditions, Godin Leisure Time Physical Activity Questionnaire, Mediterranean‐DASH Intervention for Neurodegenerative Delay (MIND) diet adherence, and cognitive outcomes, including global cognition assessed via the Mini‐Mental State Examination (MMSE) and executive function assessed via Trails A/B and verbal fluency). Risk factors (e.g., age >65, BMI >30, low education, poor sleep, inactivity, poor diet, chronic conditions) were scored dichotomously, summed, and analyzed for prevalence and clustering. Pearson's correlation tested associations between clustered risk factors and cognitive outcomes.

**Result:**

This study included 61 Latino participants (M_age_ = 58.6 ± 8.66 years, 77% women, 41.7% low income, M_BMI_ = 31.2± 5.14, M_STIC‐m_ = 36.2± 2.3). Risk factor prevalence included: 25% aged ≥65, 63.3% with ≤high school education, 55.7% sleeping <7 hours/night, 60.7% with ≥1 chronic disease, 58.8% obese, 26.2% with a MIND diet score ≤7, and 45.9% insufficiently active. Over half (61.1%) of participants had 3‐4 risk factors, 1.9% had no risk factors, and 3.7% had six. None had all seven. Clustering of risk factors correlated negatively with MMSE scores (*r* = ‐0.37, *p* = 0.005) and verbal fluency (*r* = ‐0.29, *p* = 0.035) but positively with Trails A completion time (*r* = 0.30, *p* = 0.027), but not Trails B (*r* = 0.20, *p* = 0.139).

**Conclusion:**

Increased risk factors are linked to poorer executive function and global cognition. Most participants exhibited multiple risk factors, primarily low education, poor sleep, chronic disease, and obesity. These factors are largely modifiable, underscored by the need for interventions targeting multiple risk factors to mitigate cognitive decline.

